# Concurrent physician-diagnosed asthma and chronic obstructive pulmonary disease: A population study of prevalence, incidence and mortality

**DOI:** 10.1371/journal.pone.0173830

**Published:** 2017-03-16

**Authors:** Tetyana Kendzerska, Mohsen Sadatsafavi, Shawn D. Aaron, Teresa M. To, M. Diane Lougheed, J. Mark FitzGerald, Andrea S. Gershon, for the Canadian Respiratory Research Network

**Affiliations:** 1 Ottawa Hospital Research Institute, University of Ottawa, Ottawa, ON/CA; 2 Institute for Clinical Evaluative Sciences, Ottawa, ON/CA; 3 Institute for Clinical Evaluative Sciences, Toronto, ON/CA; 4 Sunnybrook Research Institute, Toronto, ON/CA; 5 University of British Columbia, Vancouver, BC/CA; 6 University of Toronto, Toronto, ON/CA; 7 The Hospital for Sick Children, Toronto/CA; 8 Queen's University, Kingston, ON/CA; 9 Sunnybrook Health Sciences Centre, Toronto, ON/CA; Imperial College London, UNITED KINGDOM

## Abstract

**Objective:**

We conducted a population-based cohort study to estimate trends in prevalence, incidence, and mortality of concurrent physician-diagnosed asthma and chronic obstructive pulmonary disease (COPD).

**Study design and setting:**

Two validated health administrative case definitions were used to identify asthma and COPD among all individuals aged 35 years and older living in Ontario, Canada. Annual asthma, COPD, and concurrent asthma and COPD prevalence, incidence, and mortality, standardized for age and sex, were estimated, and compared from 2002 to 2012, using generalized linear models.

**Results:**

Standardized prevalence of concurrent asthma and COPD increased by 10.5%, from 2.9% in 2002 to 3.2% in 2012 overall, but more prominently in women compared to men. Overall, standardized incidence decreased by16%, from 2.5 to 2.1 per 1000 individuals, but increased significantly in young adults. All-cause mortality among patients with concurrent asthma and COPD decreased by 11.2%, from 2.6% to 2.2%. Being diagnosed with both diseases was significantly associated with higher all-cause mortality compared to asthma (OR = 1.56, 95% CI: 1.50–1.58), but not compared to COPD (OR = 0.97, 0.96–0.98), except in young adults aged 35 to 49 years where people with asthma and COPD had higher mortality (OR = 1.21, 1.15–1.27).

**Conclusions:**

In a large North American population, the burden of concurrent physician-diagnosed asthma and COPD is increasing, particularly in women and young adults.

## Introduction

In 2014, the Global Initiative for Asthma and the Global Initiative for Chronic Obstructive Lung Disease jointly published a consensus description of Asthma Chronic Obstructive Pulmonary Disease (COPD) Overlap Syndrome (ACOS) for clinical use [1]. ACOS was defined as a condition “*characterized by persistent airflow limitation with several features usually associated with asthma and several features usually associated with COPD*” [1, 2]. Patients with ACOS experience more frequent exacerbations [3–5], have a worse health-related quality of life [6], have a more rapid decline in lung function [5, 7, 8] and use more health services [9] than patients with asthma or COPD alone. Recognizing ACOS as an entity beyond the sum of its parts is important because strategies for its management are not simply those of asthma and COPD combined.

Despite the importance, many conflicting aspects in the definition and clinical significance of ACOS remain [10–13]. Some recent reviews have noted that ACOS is not a single disease entity, but rather a spectrum of diseases with many different clinical phenotypes and underlying endotypes, which might require different therapeutic target strategies [11, 12, 14]. However, before major changes in the nomenclature of disease entities will occur, it is important to generate high-quality data to pursue a better understanding of ACOS [13]. Increasing awareness of the epidemiology of ACOS within the medical community, especially over time and by sex and age, holds potential significance because the term ACOS, regardless of definition, recognizes the multitude of patients without a pure form of asthma or COPD.

The epidemiology of ACOS has not been well studied. ACOS prevalences of between 1.6% and 4.5% have been reported in the general population [5, 15–18]; however, the limited number of studies that have provided these estimates had relatively small sample sizes, did not examine ACOS epidemiology over time, and did not explore age or sex differences. There is also limited knowledge about ACOS mortality. While one might hypothesize that ACOS would have higher mortality than asthma or COPD alone because it represents a convergence of two pathological processes [5, 19], this has not been confirmed by some studies [20–23].

While the exact criteria that physicians should use to diagnose ACOS in clinical practice remain to be defined and validated, a real world, pragmatic approach to identifying individuals with ACOS is to simply recognize those who have been diagnosed with both asthma and COPD. While concurrent physician-diagnosed asthma and COPD may not reflect all phenotypes of ACOS, it can provide valuable insight into how the interaction between the two conditions affects epidemiological outcomes, such as incidence, prevalence, and mortality.

To fill some of the aforementioned gaps in ACOS epidemiology, we examined and compared prevalence trends of concurrent physician-diagnosed asthma and COPD, using the data of the entire population of a well-defined and large geographical location in North America. As secondary objectives, we also evaluated and compared incidence and mortality trends, and examined the effect of sex and age.

Some of the results of these studies have been previously reported in the form of an abstract [24].

## Materials and methods

A population-based retrospective cohort study, using universal population health administrative data housed at the Institute for Clinical Evaluative Sciences (ICES), was conducted. All individuals aged 35 years and older living in Ontario, the most populous province of Canada with a population of approximately 13 million in 2010, were considered. An age cutoff of 35 was used because COPD is uncommon below this age [25]. People with physician-diagnosed asthma, COPD, and concurrent asthma and COPD were identified in health administrative databases, using validated health administrative case definitions [26, 27]. Annual prevalence, incidence, and all-cause, respiratory- and cardiovascular-related mortality trends were estimated and compared from 2002 to 2012.

ICES has a special designation under Ontario’s Personal Health Information Protection Act (PHIPA). This means that health information custodians—like physicians, hospitals, or long-term care homes—are permitted to disclose personal health information about their patients to ICES without consent. ICES may use personal health information under the authority of PHIPA for approved research projects. A data sharing agreement with each data partner governs the privacy and security of the information in the ICES data inventory. ICES’ policies, practices, and procedures for using data are reviewed and approved on a regular basis by the Office of the Information Privacy Commissioner/Ontario. More details are available at: http://www.ices.on.ca/Data-and-Privacy/. This study was approved by the institutional review board at Sunnybrook Health Sciences Centre, Toronto, Canada. A waiver of informed consent was granted [28, 29].

### Data sources

In Ontario, details on virtually all physician and hospital services are captured in health administrative databases including: (i) the Ontario Health Insurance Plan physician services claims database; (ii) the Canadian Institute for Health Information Discharge Abstract Database; (iii) the Ontario Registered Persons Database; (iv) the Ontario Vital Stats database; (v) the Ontario Asthma Surveillance Information System [26]; and (vi) the Ontario COPD Database [27]. Details of these databases can be found on the ICES website [30]. These datasets were linked on an individual level using unique encoded identifiers. The resulting dataset used in this study is held securely in coded form at the ICES. While data sharing agreements prohibit ICES from making the dataset publicly available, access may be granted to those who meet pre-specified criteria (available at www.ices.on.ca/DAS) for confidential access. The full dataset creation plan is available from the authors upon request.

#### Definition of physician-diagnosed asthma

Details of a previously validated case definition to identify people with physician-diagnosed asthma have been described elsewhere [26, 31–33]. In brief, it consists of one asthma hospitalization or two outpatient asthma visits within two years (84% sensitivity and 76% specificity in adults when compared to a clinical reference standard which, as seen in real world practice, may or may not have included spirometry) [26, 31]. The date of the earliest asthma hospitalization or outpatient visit was used to determine the asthma diagnosis date. Individuals were considered to have *incident* asthma if they had no outpatient or inpatient asthma visits in the 5 years prior to the diagnosis date. They were otherwise categorized as having *prevalent* asthma from the date asthma first appeared in their health data until death, in order to be consistent with previous evidence indicating that asthma, once diagnosed, may remit but does not resolve [32, 34]. Patients diagnosed before age 35 (age of inclusion in this study) were considered to have prevalent asthma.

#### Definition of physician-diagnosed COPD

Details of a previously validated case definition to identify people with physician-diagnosed COPD at the time of their first COPD hospitalization or outpatient visit after 35 years of age [27] have been described elsewhere (85% sensitivity and 78% specificity when comparing with clinical evaluation, as with asthma) [27, 35–38]. As with asthma, individuals were considered to have *incident* COPD if they had no COPD hospitalization or outpatient COPD visits in the 5 years prior to their diagnosis date, otherwise they were considered to have *prevalent COPD* from the date COPD first appeared in their health data until death.

#### Definition of concurrent physician-diagnosed asthma and COPD

For individuals with both physician-diagnosed asthma and COPD, the first date they met the criteria for diagnosis of both asthma and COPD (as above) was considered their *incident date*. Because these individuals had both diseases concurrently for a period of time, we refer to them as having concurrent physician-diagnosed asthma and COPD. Individuals were considered to have *prevalent* concurrent physician-diagnosed asthma and COPD from their incident date and each year following, until death.

### Outcomes

Our primary outcome was trend in the age- and sex-standardized prevalence of concurrent asthma and COPD, between fiscal year 2002 (from April 1, 2002 to March 31, 2003) and fiscal year 2012 (from April 1, 2012, to March 31, 2013). While the data were available to 2014, our study period ended in 2012 to allow for a 2-year ‘‘look forward” period, in order to meet the terms of the asthma case definition [26, 31]. Follow-up was terminated at the earliest of date of death, moving out of the province, or the end of the study period.

Our secondary outcomes were trends in the age- and sex-standardized incidence of concurrent asthma and COPD and all-cause mortality in individuals with concurrent asthma and COPD over the same time period. All-cause mortality was chosen to eliminate potential misclassification bias associated with cause-specific mortality. Further, COPD-specific death is known to underestimate the excess overall mortality in COPD, since a significant proportion of COPD patients die of cardiovascular (CV) causes [39, 40].

### Statistical analyses

Descriptive statistics were used to characterize our population of interest. We estimated the annual prevalence of concurrent asthma and COPD by dividing the number of affected patients who were alive at the end of each fiscal year by the census population estimated in the corresponding year. A similar approach was used to calculate annual prevalence for asthma and COPD [33, 37]. The definitions of COPD, asthma, and concurrent asthma and COPD populations were not exclusive of each other.

We estimated annual concurrent asthma and COPD incidence by dividing the number of new patients in each year by the number of individuals at risk of developing concurrent asthma and COPD from census estimates in the corresponding year. A similar approach was used to calculate annual incidence for asthma and COPD [33, 37].

We estimated annual crude mortality rates by dividing the number of deaths at the end of each fiscal year among individuals with (i) prevalent asthma or (ii) prevalent COPD or (iii) prevalent, concurrent asthma and COPD by the number of individuals with each disease, respectively, in each corresponding year.

To compare trends in prevalence, incidence, and mortality rates, we standardized rates for age and sex, using 2006 Ontario census population estimates [41]. Confidence intervals (CIs) for standardized rates were calculated using a previously described method [41, 42]. Age was categorized into 35–49 (young adults), 50–64, and older than 65 years. The relative percentage changes in rates between two fiscal years were calculated using the rate in the earlier year as the reference. Generalized linear models (binomial distribution, logit link function) were used: (i) to estimate if the prevalence, incidence, and mortality of concurrent asthma and COPD had changed over time, overall (for each calendar year increase) and in people of different age groups and sex; and (ii) to determine if mortality differed between patients with asthma, COPD and concurrent asthma and COPD, controlling for age, sex and calendar time. Effects of age, sex, and calendar time on the condition of interest were tested through interactions between cohort membership, age, sex, and calendar year, respectively [37].

### Additional analyses

To adjust for potential misclassification associated with the case definition of COPD, the analyses was refitted using a definition of physician-diagnosed COPD with higher specificity, but lower sensitivity (specificity of 95%, sensitivity of 58%) [27]. According to this definition, patients aged 35 years or older had COPD as of their first COPD hospitalization or the first of three outpatient COPD visits within two years [27].

Since a significant proportion of patients with COPD die of respiratory and cardiovascular causes [39, 40], we also compared trends in mortality attributed to these causes. In order to test if differences in mortality changed when the groups were defined to be mutually exclusive, we compared mortality rates between diseases after excluding patients with concurrent asthma and COPD from the COPD and asthma populations.

All statistical analyses were performed at the ICES using R version 2.15.2 (http://www.r-project.org) and SAS 9.3.

## Results

### General characteristics of population with concurrent physician-diagnosed asthma and COPD

Among patients with asthma, 21.3% were also diagnosed with COPD. Compared to people with asthma alone, people with concurrent asthma and COPD were more likely to be female and 65 years or older. They also appeared to develop asthma at an older age than people with asthma alone (median age of 59.2 vs. 49.5 years).

Among patients with COPD, 27.6% were also diagnosed with asthma. Compared to people with COPD alone, patients with concurrent asthma and COPD were more likely to be female and below the age of 50. They also appeared to develop COPD at younger age than people with COPD alone (median age of 59.2 vs. 62.7 years).

Among patients with concurrent asthma and COPD, 30.8% had a COPD diagnosis before an asthma diagnosis and the rest asthma before COPD. The median time between COPD and asthma diagnoses (absolute difference) was 7.2 years.

### Prevalence

**Fig 1:**
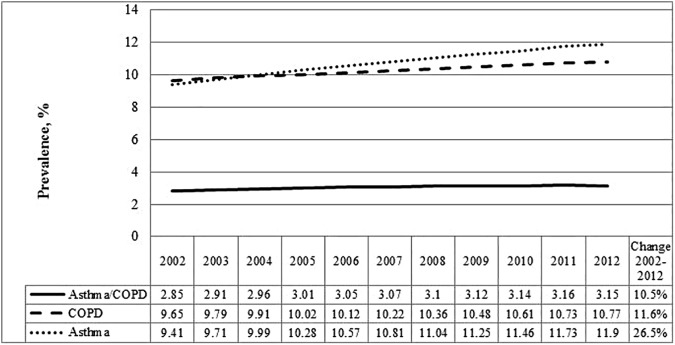
Age and sex standardized prevalence rates (%) for COPD, asthma and concurrent physician-diagnosed asthma and COPD over time among adults 35 years and older in Ontario, Canada.

In 2012, there were 7,589,414 people aged 35 years and older living in Ontario, Canada. Between 2002 and 2012, the age- and sex-standardized prevalence of concurrent asthma and COPD increased by 10.5%, from 2.9% in 2002 to 3.2% in 2012 (Fig 1, Table A in S1 Text). Female sex and older age were significantly associated with higher prevalence (Table 1). Concurrent asthma and COPD prevalence significantly increased over time in all age groups and sexes, but more prominently in women compared to men (also confirmed by a significant interaction between sex and calendar year) (Table 1).

**Table 1:** Trends in concurrent physician-diagnosed asthma and COPD prevalence, incidence and mortality over time (increase with calendar year) adjusted for sex and age in generalized linear model.

	Prevalence	Incidence	All-cause Mortality
	RR (95%CI)		
**For the entire sample** (statistical model: year, age, sex without interaction term)			
Time, increase in ten years	**1.11** (1.10–1.11)	**0.84** (0.82–0.85)	**0.88** (0.86–0.90)
Sex: male vs. female	**0.78** (0.78–0.79)	**0.79** (0.78–0.80)	**1.22** (1.21–1.24)
Age, years: 50–64 vs. 35–49	**2.82** (2.81–2.83)	**1.55** (1.53–1.56)	**2.51** (2.40–2.62)
Age, years: 65+ vs. 35–49	**6.19** (6.17–6.22)	**2.54** (2.51–2.57)	**11.87** (11.39–12.38)
**Stratified by sex controlling for age**			
**Female:** Time, change over ten years	**1.15** (1.15–1.16)	**0.84** (0.83–0.86)	**0.90** (0.87–0.92)
**Male:** Time, change over ten years	**1.04** (1.03–1.05)	**0.83** (0.81–0.85)	**0.86** (0.84–0.89)
**Stratified by age group, controlling for sex**			
**35–49 years:** Time, change over ten years	**1.12** (1.11–1.13)	**1.16** (1.13–1.20)	**0.94** (0.83–1.07)^b^
**50–64 years:** Time, change over ten years	**1.19** (1.19–1.20)	**0.87** (0.84–0.89)	**0.79** (0.75–0.84)
**65+ years:** Time, change over ten years	**1.05** (1.04–1.05)	**0.65** (0.64–0.67)	**0.89** (0.87–0.91)

Effects Reported as Relative Risks (RR)^a^ with 95% Confidence Interval (CI).

^a^The estimates were reported as relative risks instead of odds ratios because an adjusted odds ratio approximates the adjusted relative risk when disease incidence/prevalence is rare (<10%) and RR is easier to interpret.

^b^Non-significant (p values ≥0.05)

CI–confidence interval; COPD–chronic obstructive pulmonary disease; RRs—relative risks

### Incidence

**Fig 2:**
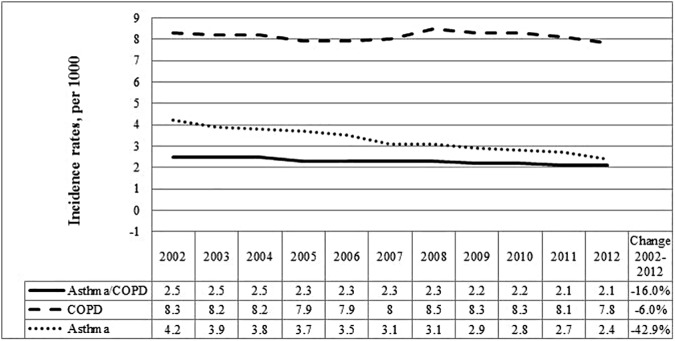
Age and sex standardized incidence rates (per 1000 individuals) for COPD, asthma and concurrent physician-diagnosed asthma and COPD over time among adults 35 years and older in Ontario, Canada.

The overall age- and sex-standardized incidence rate of concurrent asthma and COPD decreased by 16%, from 2.5 to 2.1 per 1,000 individuals from 2002 to 2012 (Fig 2, Table B in S1 Text). Female sex and older age were significantly associated with higher incidence (Table 1). Incidence significantly decreased over time in both sexes and in adults 50 years and older (Table 1). It increased, however, in young adults (also confirmed by a significant interaction between age group and calendar year).

### All-cause mortality

**Fig 3:**
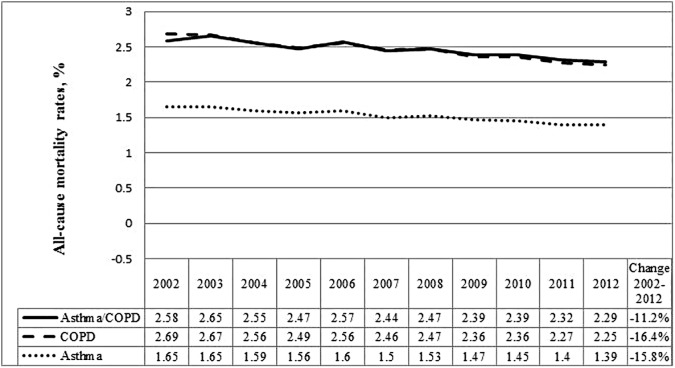
All-cause mortality standardized rates (%) by age and sex for COPD, asthma and concurrent physician-diagnosed asthma and COPD over time among adults 35 years and older in Ontario, Canada.

Overall age- and sex-standardized all-cause mortality among patients with concurrent asthma and COPD decreased by 11.2%, from 2.6% to 2.3% between 2002 and 2012 (Fig 3, Table C in S1 Text). Male sex and older age were associated with higher mortality (Table 1). Mortality decreased in both sexes and in adults 50 years and older (Table 1). It remained stable, however, in young adults (also confirmed by a significant interaction between age group and calendar year).

### Comparison of all-cause mortality between COPD, asthma and concurrent physician-diagnosed asthma and COPD

Although the magnitude of the difference was small, overall, all-cause mortality was significantly higher among patients with COPD compared to patients with concurrent asthma and COPD or asthma alone, controlling for age, sex, and calendar year (Table 2, Fig 3); however, this effect depended on age. In young adults, all-cause mortality was significantly higher in patients with concurrent asthma and COPD than COPD alone (Table 2). With concurrent asthma and COPD and COPD alone as mutually exclusive groups, similar results were obtained when comparing mortality in individuals (results not shown).

**Table 2:** The association between concurrent physician-diagnosed asthma and COPD compared to COPD or asthma with all-cause, cardiovascular and respiratory mortality rates adjusted for time (calendar year), sex, and age.

Mortality rate		Odds Ratio (95% Confidence Interval)	
		Asthma and COPD vs. asthma	Asthma and COPD vs. COPD
**All-cause mortality**			
For entire sample^a^		1.56 (1.55–1.58)	0.97 (0.96–0.98)
Model: age*condition (p<0.001)	Age 35–49	3.24 (3.09–3.40)	1.22 (1.16–1.28)
	Age 50–64	2.16 (2.05–2.28)	1.04 (0.99–1.10)^b^
	Age 65 and older	1.43 (1.36–1.50)	0.96 (0.91–1.00) ^b^
Model: sex*condition (p<0.001)	Males	1.50 (1.48–1.53)	0.98 (0.97–1.00) ^b^
	Females	1.61 (1.60–1.63)	0.96 (0.95–0.97)
**Cardiovascular-related mortality**			
For entire sample ^a^		1.47 (1.45–1.49)	0.92 (0.91–0.94)
Model: age*condition (p<0.001)	Age 35–49	3.32 (2.95–3.73)	1.24 (1.10–1.40)
	Age 50–64	2.19 (1.93–2.49)	1.07 (0.95–1.22) ^b^
	Age 65 and older	1.38 (1.22–1.55)	0.91 (0.80–1.02) ^b^
Model: sex*condition (p<0.001)	Males	1.40 (1.36–1.45)	0.95 (0.93–0.98)
	Females	1.52 (1.49–1.55)	0.89 (0.88–0.91)
**Respiratory related mortality**			
For entire sample ^a^		1.84 (1.81–1.88)	1.39 (1.37–1.41)
Model: age*condition (p<0.001)	Age 35–49	6.26 (5.37–7.30)	1.90 (1.64–2.20)
	Age 50–64	3.09 (2.62–3.64)	1.71 (1.46–2.00)
	Age 65 and older	1.71 (1.46–1.99)	1.36 (1.17–1.57)
Model: sex*condition (p<0.001)	Males	1.72 (1.65–1.78)	1.45 (1.40–1.49)
	Females	1.95 (1.90–2.00)	1.33 (1.30–1.36)

^a^ Statistical Model: year, age, sex without interaction term

^b^—non-significant (p values ≥0.05)

### Additional analysis

When we used a more specific definition of COPD, our results did not change significantly with respect to incidence and mortality trends, and differences by age and sex, for concurrent asthma and COPD (Tables D and E in S1 Text). However, unlike our main results, prevalence did not significantly increase over time.

### Cause-specific mortality

Similar to all-cause mortality, both overall respiratory- and overall CV-related mortality significantly decreased from 2002 to 2012 among patients with concurrent asthma and COPD, controlling for sex and age (Figs A and B in S1 Text; Table F in S1 Text). However, these decreases were dependent on age, with young adults having no decrease.

Concurrent asthma and COPD was significantly associated with higher CV mortality compared to asthma, but not compared to COPD, controlling for age, sex, and calendar year (Table 2). Similar to all-cause mortality, this effect depended on age, with young adults, but not older people, with concurrent asthma and COPD being more likely to die than those with COPD alone. Concurrent asthma and COPD was significantly associated with higher respiratory-related mortality compared to both asthma and COPD across all ages (Table 2).

## Discussion

We conducted a population-based study of all individuals aged 35 and older living in Ontario, Canada, between 2002 and 2012, and found that while overall prevalence of concurrent physician-diagnosed asthma and COPD increased (or stayed stable in some subgroups), its overall incidence and all-cause mortality decreased. We also found that prevalence in women and young adults increased more than in men and older adults. Young adults also experienced increases in incidence with no notable improvement in mortality over time. To the best of our knowledge, this is the most detailed, large-scale, population-based study of concurrent asthma and COPD epidemiology, and it fills many gaps in knowledge on the overlap between these two diseases, including its age and sex distribution. Our results reveal that the burden of concurrent asthma and COPD is substantial and likely to escalate, due to its increasing prevalence and impact on young adults destined to live with it for the rest of their lives. This is especially concerning given its worse prognosis, more frequent symptoms, worse quality of life, and greater comorbidity burden compared to COPD alone [3–6]. Knowing the anticipated increasing burden of concurrent asthma and COPD, this will allow health care providers and policy makers to plan accordingly. Our results also help identify which high-risk groups are most affected.

The prevalence rates for concurrent asthma and COPD found in our study are comparable with or slightly higher than other population prevalence rates reported for asthma-COPD overlap, even though we used different definitions than those studies (i.e. self-reported, based on ICD-codes, and using instrumental measures and clinical symptoms) [5, 15–18]. Similarly, our prevalence rates, as that of ACOS studies, increased with age [15, 17] and was higher in women compared to men [15, 17, 18]. Finally, the age distribution of concurrent asthma and COPD patients found in our study was also similar to previous studies [5, 6, 16, 18], where individuals with concurrent asthma and COPD were younger than COPD patients and older than asthma patients.

The upward trend of concurrent asthma and COPD age- and sex-standardized prevalence may be explained by increasing prevalence of both asthma and COPD [33, 37, 43–45] (Fig 1). Of note, standardization for age means our results cannot be attributed to the aging of the population. The more pronounced increase in concurrent asthma and COPD in women is consistent with other studies [3, 46] and may be explained by a greater increase in COPD prevalence, over time, in women compared to men [37, 47], a higher prevalence of asthma in women 35 years and older than in men of the same age [33, 43], and a slower decline in smoking combined with a stronger negative impact of smoking in women compared to men [48, 49]. These differences between the sexes is deserving of further investigation.

Similar to prevalence, decrease in concurrent asthma and COPD incidence may be explained by decreases in both asthma and COPD incidence [33, 37, 45]. Importantly, we found that incidence significantly increased over time in young adults. This group also did not experience improvements in mortality, as older adults did, and had higher all- and CV-related mortality compared to people with COPD. Accordingly, young adults appear to be at higher risk of developing ACOS. As such, this group, who is also more likely to develop COPD from chronic severe childhood asthma, should be considered for targeted prevention and management [50].

Overall decrease in age- and sex-standardized all-cause mortality rates among patients with concurrent asthma and COPD may be explained by decreases in mortality of both COPD and asthma, over time [33, 37, 43, 51]. It is also consistent with all-cause mortality trends in the Canadian population [52]. Our findings of a more pronounced decrease in men compared to women is similar to findings in the COPD population [37] and consistent with patterns in the general population [53].

Similar to previous, relatively small longitudinal studies, older people with COPD had higher mortality than older people with asthma or concurrent asthma and COPD [20, 21, 23]. This may be because COPD without a concurrent diagnosis of asthma, is more likely to develop from smoking than from chronic asthma—the former being associated with a higher mortality than the latter, due to other consequences, such as cancer or CV disease [54]. In younger people, as mentioned above, concurrent asthma and COPD was associated with higher mortality than COPD. This is consistent with a previous population-based study of young adults that found that those with both asthma and COPD had more hospitalizations and worse lung function than individuals with asthma or COPD alone [55]. Diagnosis of concurrent asthma and COPD at younger age may reflect a subtype of the disease with more rapidly declining lung function and worse prognosis than other subtypes [55, 56].

The strengths of our study are the use of large comprehensive health administrative databases from an entire provincial population to identify individuals with physician-diagnosed asthma and COPD, its use of validated algorithms, its ability to assess age and sex differences, and to follow people over many years.

Our study had limitations which merit emphasis. In the absence of a validated health administrative data definition of ACOS, we studied concurrent asthma and COPD using validated case definitions of asthma and COPD. Development of an ACOS health administrative definition is an important area of future study, once consensus regarding the clinical definition has been reached [1]. We were reassured, however, by the similar characteristics of our concurrent asthma and COPD population and those of previous ACOS studies [3, 35, 57, 58]. Still, we cannot rule out ‘diagnostic exchange’ as a potential source of error, whereby an individual physician might have misattributed respiratory symptoms of COPD as asthma, or vice-versa. These non-directional errors, however, likely caused random variation or noise, but not bias that affected trends, especially given the generally long interval between the two diagnoses (average 7.6 years). They might have also led to the conservative underestimation of concurrent asthma and COPD’s large health care burden, if physicians were dissuaded from looking for a second diagnosis after a first was made. Alternatively, it is possible that patients with more severe asthma or COPD were more likely to receive additional diagnosis (asthma or COPD on top of a previous COPD or asthma diagnosis) because they had more contact with physicians. This could have led to an overestimate of ACOS and/or an overestimate of its health care burden.

We were also reassured by the confirmation of our findings when an alternative, more specific case definition of COPD was used. When using this definition, however, we did not see an increase in concurrent asthma and COPD prevalence over time. This is probably because the specific definition detected more severe COPD cases whose prevalence has been relatively stable [59], likely due to decreasing smoking rates [60] and improvements in management. Conversely, the specific COPD case definition likely missed people with milder COPD and underestimated the true burden of concurrent asthma and COPD. The specific definition results combined with those from the main analysis provide upper and lower limits within which true concurrent asthma and COPD prevalence, incidence, and mortality likely lie.

A second limitation was that we were only able to study physician-diagnosed and not spirometry-confirmed concurrent asthma and COPD. This is because only 30 to 50% of people in Ontario complete spirometry before being diagnosed with COPD or asthma [38, 61]. Without spirometry, physicians are known to miss milder disease and underdiagnose COPD. This could have led us to underestimate prevalence and incidence [62]. Indeed, our prevalence estimates were similar or slightly lower than ACOS estimates obtained using spirometry [5, 6, 55]. However, while some people with mild disease might have been missed, our findings are generalizable to real-world patients responsible for the largest burden of concurrent asthma and COPD, in terms of health care resources—the ones that clinicians and policy makers are usually most concerned about. Also, any underestimate was likely consistent over time and unlikely to affect trends.

## Conclusion

In conclusion, we conducted an analysis using comprehensive health administrative data on a large North American population. We found that the overall prevalence of concurrent physician-diagnosed asthma and COPD increased, its overall incidence and all-cause mortality decreased, and that women and younger adults experienced a significantly greater burden. Our results are important to ensure health care providers and policy makers are prepared for the increasing burden of concurrent asthma and COPD and know which high risk groups will be most affected. Future research should focus on validating a health administrative case definition of ACOS, confirming these findings in other populations, and identifying high-risk groups for whom specific preventive and management strategies may be required.

## Supporting information

S1 Text Online data supplement. (DOCX)
