# Supplementary material for: Concurrent physician-diagnosed asthma and chronic obstructive pulmonary disease: A population study of prevalence, incidence and mortality
Source: PLoS One. 2017 Mar 16;12(3):e0173830. doi: 10.1371/journal.pone.0173830 (PMC5354414; doi:10.1371/journal.pone.0173830)
Supplement: S1 Text — (DOCX) [file pone.0173830.s001.docx]

**Online Data Supplement**

**Title:** Concurrent Physician-Diagnosed Asthma and Chronic Obstructive Pulmonary Disease: A Population-Based Cohort Study of Prevalence, Incidence and Mortality in Ontario, Canada.

Kendzerska T., Sadatsafavi M., Aaron S.D., To T., Lougheed M.D., FitzGerald J M., Gershon A., for the Canadian Respiratory Research Network.

**Tables:**

**Table A.** Standardized and crude prevalence rates (%) for concurrent physician-diagnosed asthma and COPD overtime among adults 35 years and older in Ontario, Canada.

**Table B**. Standardized and crude incidence rates (per 1000 individuals) for concurrent physician-diagnosed asthma and COPD over time among adults 35 years and older in Ontario, Canada.

**Table C**. Standardized and crude all-cause mortality rates (%) for concurrent physician-diagnosed asthma and COPD over time among adults 35 years and older in Ontario, Canada.

**Table D**. Changes in concurrent physician-diagnosed asthma and COPD (specific case definition of COPD) prevalence, incidence and mortality over time adjusted for sex and age.

**Table E**. Effect of concurrent physician-diagnosed asthma and COPD compared to COPD (specific case definition of COPD) or asthma on mortality rates (all and specific causes) adjusted for time, sex, and age.

**Table F**. Trends in concurrent physician-diagnosed asthma and COPD cardiovascular- and respiratory-related mortality rates over time (increase with calendar year) adjusted for sex and age.

**Figures**:

**Figure A**. Age and sex respiratory-related mortality standardized rates (%) for COPD, asthma and concurrent physician-diagnosed asthma and COPD over time among adults 35 years and older in Ontario, Canada.

**Figure B**. Age and sex cardiovascular-related mortality standardized rates (%) for COPD, asthma and concurrent physician-diagnosed asthma and COPD over time among adults 35 years and older in Ontario, Canada.

**Table A.** Standardized and crude prevalence rates (%) for concurrent physician-diagnosed asthma and COPD overtime among adults 35 years and older in Ontario, Canada.

| **Year** | **Age group** | **Overall** | | | **Females** | | | **Males** | | |
| --- | --- | --- | --- | --- | --- | --- | --- | --- | --- | --- |
|  |  | # with asthma  /COPD | Population | Standardized rate, %  (95%CI) | # with asthma  /COPD | Population | Crude rate, %  (95%CI) | # with  asthma  /COPD | Population | Crude rate, %  (95%CI) |
| **2002** | **35-49** | 31,041 | 2,977,984 | 1.05 (1.04-1.06) | 18,840 | 1,488,684 | 1.27 (1.25-1.28) | 12,201 | 1,489,300 | 0.82 (0.80-0.83) |
|  | **50-64** | 54,170 | 1,950,408 | 2.79 (2.76-2.81) | 32,213 | 989,588 | 3.26 (3.22-3.29) | 21,957 | 960,820 | 2.29 (2.26-2.32) |
|  | **65+** | 95,721 | 1,521,218 | 6.29 (6.25-6.33) | 54,484 | 864,597 | 6.30 (6.25-6.35) | 41,237 | 656,621 | 6.28 (6.22-6.34) |
|  | **all** | **180,932** | **6,449,610** | **2.85 (2.84-2.86)#** | **105,537** | **3,342,869** | **3.11 (3.09-3.13)*** | **75,395** | **3,106,741** | **2.59 (2.57-2.61)*** |
| **2004** | **35-49** | 32,800 | 3,028,744 | 1.09 (1.08-1.10) | 19,831 | 1,509,089 | 1.31 (1.30-1.33) | 12,969 | 1,519,655 | 0.85 (0.84-0.87) |
|  | **50-64** | 60,928 | 2,101,104 | 2.91 (2.89-2.93) | 36,552 | 1,068,326 | 3.42 (3.39-3.46) | 24,376 | 1,032,778 | 2.36 (2.33-2.39) |
|  | **65+** | 103,368 | 1,584,963 | 6.51 (6.47-6.55) | 59,612 | 899,941 | 6.62 (6.57-6.68) | 43,756 | 685,022 | 6.39 (6.33-6.45) |
|  | **all** | **197,096** | **6,714,811** | **2.96 (2.95-2.98)#** | **115,995** | **3,477,356** | **3.26 (3.24-3.28)*** | **81,101** | **3,237,455** | **2.66 (2.64-2.67)*** |
| **2006** | **35-49** | 33,318 | 3,052,920 | 1.10 (1.09-1.11) | 20,063 | 1,517,346 | 1.32 (1.30-1.34) | 13,255 | 1,535,574 | 0.86 (0.85-0.88) |
|  | **50-64** | 68,516 | 2,260,636 | 3.04 (3.02-3.06) | 41,340 | 1,148,902 | 3.60 (3.56-3.63) | 27,176 | 1,111,734 | 2.44 (2.42-2.47) |
|  | **65+** | 110,392 | 1,653,198 | 6.66 (6.62-6.69) | 64,351 | 936,493 | 6.87 (6.82-6.92) | 46,041 | 716,705 | 6.42 (6.37-6.48) |
|  | **all** | **212,226** | **6,966,754** | **3.05 (3.03-3.06)#** | **125,754** | **3,602,741** | **3.38 (3.36-3.40)*** | **86,472** | **3,364,013** | **2.7 (2.68-2.71)*** |
| **2008** | **35-49** | 33,916 | 3,033,574 | 1.13 (1.11-1.14) | 20,468 | 1,512,093 | 1.35 (1.34-1.37) | 13,448 | 1,521,481 | 0.88 (0.87-0.90) |
|  | **50-64** | 75,989 | 2,418,182 | 3.15 (3.13-3.18) | 46,018 | 1,228,781 | 3.75 (3.71-3.78) | 29,971 | 1,189,401 | 2.52 (2.49-2.55) |
|  | **65+** | 116,726 | 1,741,246 | 6.67 (6.63-6.71) | 68,691 | 980,071 | 7.01 (6.96-7.06) | 48,035 | 761,175 | 6.31 (6.25-6.37) |
|  | **all** | **226,631** | **7,193,002** | **3.10 (3.09-3.11)#** | **135,177** | **3,720,945** | **3.47 (3.45-3.49)*** | **91,454** | **3,472,057** | **2.70 (2.68-2.72)*** |
| **2010** | **35-49** | 34,474 | 2,991,948 | 1.16 (1.15-1.17) | 20,713 | 1,499,201 | 1.38 (1.36-1.40) | 13,761 | 1,492,747 | 0.92 (0.91-0.94) |
|  | **50-64** | 83,598 | 2,581,836 | 3.25 (3.23-3.27) | 50,702 | 1,310,666 | 3.87 (3.83-3.90) | 32,896 | 1,271,170 | 2.59 (2.56-2.62) |
|  | **65+** | 122,976 | 1,841,782 | 6.64 (6.60-6.68) | 73,010 | 1,030,505 | 7.08 (7.03-7.14) | 49,966 | 811,277 | 6.16 (6.11-6.21) |
|  | **all** | **241,048** | **7,415,566** | **3.14 (3.13-3.15)#** | **144,425** | **3,840,372** | **3.54 (3.52-3.56)*** | **96,623** | **3,575,194** | **2.71 (2.69-2.72)*** |
| **2012** | **35-49** | 33,581 | 2,882,550 | 1.17 (1.16-1.18) | 20,143 | 1,456,995 | 1.38 (1.36-1.40) | 13,438 | 1,425,555 | 0.94 (0.93-0.96) |
|  | **50-64** | 90,033 | 2,734,532 | 3.30 (3.28-3.33) | 54,335 | 1,388,081 | 3.91 (3.88-3.95) | 35,698 | 1,346,451 | 2.65 (2.62-2.68) |
|  | **65+** | 130,464 | 1,972,332 | 6.57 (6.53-6.61) | 78,061 | 1,096,824 | 7.12 (7.07-7.17) | 52,403 | 875,508 | 5.99 (5.93-6.04) |
|  | **all** | **254,078** | **7,589,414** | **3.15 (3.14-3.16)#** | **152,539** | **3,941,900** | **3.56 (3.55-3.58)*** | **101,539** | **3,647,514** | **2.69 (2.68-2.71)*** |

* Age standardized rate

# Age and sex standardized rate

COPD – chronic obstructive pulmonary disease

**Table B.** Standardized and crude incidence rates (per 1000 individuals) for concurrent physician-diagnosed asthma and COPD over time among adults 35 years and older in Ontario, Canada.

| **Year** | **Age group** | **Overall** | | | **Females** | | | **Males** | | |
| --- | --- | --- | --- | --- | --- | --- | --- | --- | --- | --- |
|  |  | # with new asthma/COPD | Population | Standardized rate  (per 1000), 95%CI | # with new asthma/COPD | Population | Crude rate  (per 1000), 95%CI | # with new asthma/COPD | Population | Crude rate  (per 1000), 95%CI |
| **2002** | **35-49** | 3,986 | 2,977,984 | 1.3 (1.3-1.4) | 2,434 | 1,488,684 | 1.6 (1.6-1.7) | 1,552 | 1,489,300 | 1.0 (1.0-1.1) |
|  | **50-64** | 4,877 | 1,950,408 | 2.5 (2.4-2.6) | 2,845 | 989,588 | 2.9 (2.8-3.0) | 2,032 | 960,820 | 2.1 (2.0-2.2) |
|  | **65+** | 7,186 | 1,521,218 | 4.7 (4.6-4.8) | 4,119 | 864,597 | 4.8 (4.6-4.9) | 3,067 | 656,621 | 4.7 (4.5-4.8) |
|  | **all** | **16,049** | **6,449,610** | **2.5 (2.5-2.6)#** | **9,398** | **3,342,869** | **2.8 (2.7-2.8)*** | **6,651** | **3,106,741** | **2.3 (2.2-2.3)*** |
| **2004** | **35-49** | 4,347 | 3,028,744 | 1.4 (1.4-1.5) | 2,638 | 1,509,089 | 1.7 (1.7-1.8) | 1,709 | 1,519,655 | 1.1 (1.1-1.2) |
|  | **50-64** | 5,047 | 2,101,104 | 2.4 (2.3-2.5) | 2,985 | 1,068,326 | 2.8 (2.7-2.9) | 2,062 | 1,032,778 | 2.0 (1.9-2.1) |
|  | **65+** | 7,011 | 1,584,963 | 4.4 (4.3-4.5) | 4,024 | 899,941 | 4.5 (4.3-4.6) | 2,987 | 685,022 | 4.4 (4.2-4.5) |
|  | **all** | **16,405** | **6,714,811** | **2.5 (2.4-2.5)#** | **9,647** | **3,477,356** | **2.7 (2.7-2.8)*** | **6,758** | **3,237,455** | **2.2 (2.1-2.2)*** |
| **2006** | **35-49** | 4,343 | 3,052,920 | 1.4 (1.4-1.5) | 2,607 | 1,517,346 | 1.7 (1.7-1.8) | 1,736 | 1,535,574 | 1.1 (1.1-1.2) |
|  | **50-64** | 5,133 | 2,260,636 | 2.3 (2.2-2.3) | 3,003 | 1,148,902 | 2.6 (2.5-2.7) | 2,130 | 1,111,734 | 1.9 (1.8-2.0) |
|  | **65+** | 6,756 | 1,653,198 | 4.1 (4.0-4.2) | 3,817 | 936,493 | 4.1 (3.9-4.2) | 2,939 | 716,705 | 4.1 (4.0-4.3) |
|  | **all** | **16,232** | **6,966,754** | **2.3 (2.3-2.4)#** | **9,427** | **3,602,741** | **2.6 (2.5-2.6)*** | **6,805** | **3,364,013** | **2.1 (2.0-2.1)*** |
| **2008** | **35-49** | 4,822 | 3,033,574 | 1.6 (1.6-1.6) | 2,922 | 1,512,093 | 1.9 (1.9-2.0) | 1,900 | 1,521,481 | 1.2 (1.2-1.3) |
|  | **50-64** | 5,578 | 2,418,182 | 2.3 (2.3-2.4) | 3,275 | 1,228,781 | 2.7 (2.6-2.8) | 2,303 | 1,189,401 | 1.9 (1.9-2.0) |
|  | **65+** | 6,064 | 1,741,246 | 3.5 (3.4-3.6) | 3,520 | 980,071 | 3.6 (3.5-3.7) | 2,544 | 761,175 | 3.3 (3.2-3.5) |
|  | **all** | **16,464** | **7,193,002** | **2.3 (2.2-2.3)#** | **9,717** | **3,720,945** | **2.6 (2.5-2.6)*** | **6,747** | **3,472,057** | **2.0 (1.9-2.0)*** |
| **2010** | **35-49** | 4,806 | 2,991,948 | 1.6 (1.6-1.7) | 2,854 | 1,499,201 | 1.9 (1.8-2.0) | 1,952 | 1,492,747 | 1.3 (1.3-1.4) |
|  | **50-64** | 5,774 | 2,581,836 | 2.2 (2.2-2.3) | 3,379 | 1,310,666 | 2.6 (2.5-2.7) | 2,395 | 1,271,170 | 1.9 (1.8-2.0) |
|  | **65+** | 6,179 | 1,841,782 | 3.3 (3.3-3.4) | 3,589 | 1,030,505 | 3.5 (3.4-3.6) | 2,590 | 811,277 | 3.2 (3.1-3.3) |
|  | **all** | **16,759** | **7,415,566** | **2.2 (2.2-2.3)#** | **9,822** | **3,840,372** | **2.5 (2.4-2.5)*** | **6,937** | **3,575,194** | **1.9 (1.9-2.0)*** |
| **2012** | **35-49** | 4,384 | 2,882,550 | 1.5 (1.5-1.6) | 2,591 | 1,456,995 | 1.8 (1.7-1.8) | 1,793 | 1,425,555 | 1.3 (1.2-1.3) |
|  | **50-64** | 5,669 | 2,734,532 | 2.1 (2.0-2.1) | 3,248 | 1,388,081 | 2.3 (2.3-2.4) | 2,421 | 1,346,451 | 1.8 (1.7-1.9) |
|  | **65+** | 6,216 | 1,972,332 | 3.1 (3.1-3.2) | 3,638 | 1,096,824 | 3.3 (3.2-3.4) | 2,578 | 875,508 | 2.9 (2.8-3.1) |
|  | **all** | **16,269** | **7,589,414** | **2.1 (2.1-2.1)#** | **9,477** | **3,941,900** | **2.3 (2.3-2.4)*** | **6,792** | **3,647,514** | **1.8 (1.8-1.9)*** |

* Age standardized rate

# Age and sex standardized rate

COPD – chronic obstructive pulmonary disease

**Table C.** Standardized and crude all-cause mortality rates (%) for concurrent physician-diagnosed asthma and COPD over time among adults 35 years and older in Ontario, Canada.

| **Year** | **Age group** | **Overall** | | | **Females** | | | **Males** | | |
| --- | --- | --- | --- | --- | --- | --- | --- | --- | --- | --- |
|  |  | # of death | Population  with asthma/COPD | Standardized rate, % (95%CI) | # of death | Population  with asthma/COPD | Crude rate, %  (95%CI) | # of death | Population  with asthma/COPD | Crude rate, %  (95%CI) |
| **2002** | **35-49** | 182 | 31,041 | 0.61 (0.52-0.71) | 90 | 18,840 | 0.48 (0.38-0.59) | 92 | 12,201 | 0.75 (0.61-0.92) |
|  | **50-64** | 969 | 54,170 | 1.83 (1.72-1.95) | 500 | 32,213 | 1.55 (1.42-1.69) | 469 | 21,957 | 2.14 (1.95-2.34) |
|  | **65+** | 6,924 | 95,721 | 7.31 (7.13-7.48) | 3,616 | 54,484 | 6.64 (6.42-6.86) | 3,308 | 41,237 | 8.02 (7.75-8.30) |
|  | **all** | **8,075** | **180,932** | **2.58 (2.52-2.65)** | **4,206** | **105,537** | **2.29 (2.21-2.37)*** | **3,869** | **75,395** | **2.93 (2.82-3.04)*** |
| **2004** | **35-49** | 224 | 32,800 | 0.71 (0.61-0.81) | 115 | 19,831 | 0.58 (0.48-0.70) | 109 | 12,969 | 0.84 (0.69-1.01) |
|  | **50-64** | 972 | 60,928 | 1.66 (1.55-1.77) | 471 | 36,552 | 1.29 (1.17-1.41) | 501 | 24,376 | 2.06 (1.88-2.24) |
|  | **65+** | 7,383 | 103,368 | 7.22 (7.05-7.38) | 3,949 | 59,612 | 6.62 (6.42-6.83) | 3,434 | 43,756 | 7.85 (7.59-8.12) |
|  | **all** | **8,579** | **197,096** | **2.55 (2.48-2.62)** | **4,535** | **115,995** | **2.24 (2.17-2.32)*** | **4,044** | **81,101** | **2.90 (2.79-3.01)*** |
| **2010** | **35-49** | 237 | 34,474 | 0.70 (0.61-0.79) | 132 | 20,713 | 0.64 (0.53-0.76) | 105 | 13,761 | 0.76 (0.62-0.92) |
|  | **50-64** | 1,181 | 83,598 | 1.44 (1.36-1.52) | 658 | 50,702 | 1.30 (1.20-1.40) | 523 | 32,896 | 1.59 (1.46-1.73) |
|  | **65+** | 8,365 | 122,976 | 6.87 (6.73-7.03) | 4,686 | 73,010 | 6.42 (6.24-6.60) | 3,679 | 49,966 | 7.36 (7.13-7.60) |
|  | **all** | **9,783** | **241,048** | **2.39 (2.34-2.46)** | **5,476** | **144,425** | **2.22 (2.15-2.30)*** | **4,307** | **96,623** | **2.60 (2.50-2.70)*** |
| **2012** | **35-49** | 178 | 33,581 | 0.54 (0.47-0.63) | 93 | 20,143 | 0.46 (0.37-0.57) | 85 | 13,438 | 0.63 (0.51-0.78) |
|  | **50-64** | 1,278 | 90,033 | 1.45 (1.37-1.54) | 688 | 54,335 | 1.27 (1.17-1.36) | 590 | 35,698 | 1.65 (1.52-1.79) |
|  | **65+** | 8,616 | 130,464 | 6.70 (6.56-6.84) | 4,791 | 78,061 | 6.14 (5.96-6.31) | 3,825 | 52,403 | 7.30 (7.07-7.53) |
|  | **all** | **10,072** | **254,078** | **2.29 (2.23-2.34)** | **5,572** | **152,539** | **2.07 (2.00-2.14)*** | **4,500** | **101,539** | **2.55 (2.45-2.64)*** |

COPD – chronic obstructive pulmonary disease

**Table D**. Changes in concurrent physician-diagnosed asthma and COPD prevalence, incidence and mortality over time adjusted for sex and age (using COPD case specific definition). Effects reported as relative risks (RR)* with 95% confidence interval (CI).

|  | **Prevalence**  **RR (95%CI)** | **Incidence**  **RR (95%CI)** | **All-cause Mortality**  **RR (95%CI)** |
| --- | --- | --- | --- |
| **For the entire sample** | | | |
| Time, increase in ten years | 0.92 (0.92-0.93) | 0.73 (0.71-0.74) | 0.97 (0.94-0.99) |
| Gender: male vs. female | 0.90 (0.90-0.90) | 0.89 (0.88- 0.90) | 1.15 (1.14-1.17) |
| Age, years:  50-64 vs. 35-49 | 4.52 (4.49- 4.55) | 2.93 (2.87-2.99) | 2.05 (1.93-2.18) |
| Age, years:  65+ vs. 35-49 | 14.84 (14.74-14.94) | 7.13 (7.00- 7.26) | 7.42 (7.02- 7.85) |
| **Stratified by sex controlling for age** | | | |
| **Female:** Time, increase in ten years | 0.98 (0.97-0.99) | 0.75 (0.73-0.76) | **0.98 (0.95-1.01)** |
| **Male:** Time, increase in ten years | 0.85 (0.84-0.86) | 0.70 (0.68-0.72) | 0.95 (0.92-0.98) |
| **Stratified by age group controlling for sex** | | | |
| **35-49 years:** Time, increase in ten years | 0.92 (0.90-0.94) | **0.99 (0.94-1.04)** | **1.11 (0.93-1.32)** |
| **50-64 years:** Time, increase in ten years | 0.98 (0.97-0.99) | 0.80 (0.77-0.82) | 0.91 (0.85-0.97) |
| **65+ years:** Time, increase in ten years | 0.90 (0.89-0.91) | 0.64 (0.62-0.66) | 0.97 (0.95-1.00) |

*The estimates were reported as RRs because an adjusted odds ratio approximates the adjusted relative risk when disease incidence/prevalence is rare (<10%) and RR is easier to interpret.

COPD – chronic obstructive pulmonary disease; RRs - relative risks

**Table E**. Effect of concurrent physician-diagnosed asthma and COPD compared to COPD (specific case definition of COPD) or asthma on mortality rates (all and specific causes) adjusted for time, sex, and age.

| **Mortality rate** | | **OR (95% CI)** | |
| --- | --- | --- | --- |
|  |  | **ACOS vs. asthma** | **ACOS vs. COPD** |
| **All-cause mortality** | | | |
| For entire sample* | | 2.14 (2.12-2.16) | 0.89 (0.88-0.90) |
| Model: age*condition (p≤0.001) | Age group 35-49 | 6.79 (6.39-7.21) | **1.01 (0.94-1.08)** |
|  | Age group 50-64 | 3.72 (3.48-3.97) | 0.90 (0.83-0.96) |
|  | Age group 65+ | 1.88 (1.77-2.00) | 0.88 (0.83-0.95) |
| Model: sex*condition (sex*asthma, p<0.0001; sex*COPD, p= 0.678) | Males | 1.98 (1.95-2.02) | 0.89 (0.87-0.90) |
|  | Females | 2.27 (2.24-2.30) | 0.89 (0.88-0.90) |
| **Circulatory related mortality** | | | |
| For entire sample* | | 1.90 (1.87-1.93) | 0.86 (0.85-0.87) |
| Model: age*condition (p≤0.03) | Age group 35-49 | 7.26 (6.25-8.43) | **1.13 (0.96-1.33)** |
|  | Age group 50-64 | 3.75 (3.20-4.39) | **0.93 (0.78-1.11)** |
|  | Age group 65+ | 1.72 (1.48-2.01) | **0.85 (0.72-1.01)** |
| Model: sex*condition (sex*asthma, p<0.0001; sex*COPD, p= 0.07) | Males | 1.75 (1.70-1.81) | 0.87 (0.84-0.90) |
|  | Females | 2.03 (1.98-2.07) | 0.85 (0.83-0.86) |
| **Respiratory related mortality** | | | |
| For entire sample* | | 2.85 (2.80-2.91) | 1.14 (1.12-1.16) |
| Model: age*condition  (age*asthma, p<0.0001;) (p<0.001) | Age group 35-49 | 19.50 (16.57-22.96) | 1.35 (1.14-1.59) |
|  | Age group 50-64 | 6.58 (5.52-7.83) | 1.25 (1.05-1.49) |
|  | Age group 65+ | 2.50 (2.12-2.95) | 1.13 (0.95-1.33) |
| Model: sex*condition (p<0.001) | Males | 2.52 (2.43-2.62) | 1.17 (1.14-1.21) |
|  | Females | 3.14 (3.06-3.22) | 1.10 (1.08-1.13) |

*Statistical Model: year, age, gender without interaction term

**Table F**. Trends in concurrent physician-diagnosed asthma and COPD cardiovascular- and respiratory-related mortality rates over time (increase with calendar year) adjusted for sex and age. Effects reported as relative risks (RR)* with 95% confidence interval (CI).

|  | **Circulatory related mortality** | **Respiratory related mortality** |
| --- | --- | --- |
|  | **RR (95%CI)** | |
| **For the entire sample (statistical model: year, age, sex without interaction term)** | | |
| Time, increase in ten years | **0.95**  (0.95-0.96) | **0.97**  (0.97-0.98) |
| **Stratified by sex controlling for age** | | |
| **Female**: Time, increase in ten years | **0.95**  (0.95-0.96) | **0.97**  (0.97-0.98) |
| **Male**: Time, increase in ten years | **0.95**  (0.94-0.96) | **0.97**  (0.96-0.98) |
| **Stratified by age group, controlling for sex** | | |
| 35-49 years: Time, increase in ten years | **0.99**  (0.96-1.02) | **0.99**  (0.96-1.03), |
| 50-64 years: Time, increase in ten years | **0.95**  (0.94-0.96) | **0.96**  (0.95-0.98) |
| 65+ years: Time, increase in ten years | **0.95**  (0.95-0.96) | **0.97**  (0.97-0.98) |

**Figure A.** Age and sex respiratory-related mortality standardized rates (%) for COPD, asthma and concurrent physician-diagnosed asthma and COPD over time among adults 35 years and older in Ontario, Canada.

**Figure B.** Age and sex cardiovascular-related mortality standardized rates (%) for COPD, asthma and concurrent physician-diagnosed asthma and COPD over time among adults 35 years and older in Ontario, Canada.
